# Co-design of a digital health intervention to provide pharmacy-based, person-centered contraceptive decision support for youth in Kenya

**DOI:** 10.1371/journal.pgph.0007335

**Published:** 2026-09-17

**Authors:** Elizabeth K. Harrington, Lisa Orii, Nelson K. Cheruiyot, Merceline Awuor, Cellestine Aoko, Dismas C. Ouma, Christine Dehlendorf, Richard Anderson, Elizabeth A. Bukusi, Serah W. Gitome

**Affiliations:** 1 Department of Obstetrics and Gynecology, University of Washington, Seattle, Washington, United States of America; 2 Department of Global Health, University of Washington, Seattle, Washington, United States of America; 3 Paul G. Allen School of Computer Science and Engineering, University of Washington, Seattle, Washington, United States of America; 4 Independent Design Consultant, Nairobi, Kenya; 5 Centre for Microbiology Research, Kenya Medical Research Institute, Nairobi, Kenya; 6 Department of Family and Community Medicine, University of California, San Francisco, San Francisco, California, United States of America; Durban University of Technology, SOUTH AFRICA

## Abstract

Adolescent girls and young women (AGYW; age 15–24 years) face intersectional barriers to undesired pregnancy prevention, with profound health consequences. Community pharmacies remain a relatively untapped opportunity to improve the reach and quality of contraceptive care for youth. The purpose of this study was to co-design and prototype a digital health, person-centered contraceptive decision support application (the Mara Divas App) tailored for Kenyan AGYW. We engaged AGYW with recent experience seeking contraception in a pharmacy, and pharmacy providers who dispense contraceptive methods, to participate in full-day human-centered design workshops. The design team analyzed visual artifacts, official written notes, and transcripts to generate insights, which were used by the technical team to modify the prototype between and after workshops. We conducted four co-design workshops among AGYW (n = 33), and pharmacy providers (n = 10). Journey mapping highlighted AGYW anxiety rooted in social stigma throughout their pharmacy experience and limited contraceptive knowledge. Friendly, nonjudgmental providers and respect for privacy were critical to engaging AGYW despite their tendency to avoid interaction and make purchases at “rocket speed.” Emergency contraception seekers need a “hook” motivating them to engage with the app, as they have low motivation to engage in contraceptive counseling due to perceived limited options. All content could be engaged in three languages by screen; AGYW switched languages frequently during app use. AGYW preferred video and visual content to text, though audio narration of text was popular. Additional features were added to enhance navigability, which was initially limited among the youngest participants. AGYW desired the app to project a confident, powerful “diva.” We used human-centered design approaches to generate key design insights related to engaging youth seeking emergency contraception in pharmacies, overcoming literacy challenges with multilingual audiovisual content, and addressing negative contraceptive beliefs. Future research is needed to study the feasibility and determinants of app implementation.

## Introduction

Autonomy over decisions about whether and when to have a child is essential to achieving gender equity and reducing maternal mortality (Sustainable Development Goals 5, 3.1 [1]). While undesired pregnancy prevention among adolescent girls and young women (AGYW; age 15–24 years) is an established human rights and global health priority, youth experience structural vulnerability rooted in poverty, gender inequality, and differential access to sexual and reproductive (SRH) education and services, including contraception [2–6]. In East Africa, over half of pregnancies among adolescents are estimated to be “unintended [7] (63% in Kenya [8]. Undesired pregnancies may lead to profound health consequences [9–11] at a critical time in young people’s psychosocial and physical development [12,13]. Kenyan AGYW face community- and health systems-level stigma related to sexuality and contraceptive use [14,15], fueling pervasive concerns about future fertility consequences [5] and compounding country-level policy challenges [4]. In recent years, AGYW have been a priority population for the global family planning community [16,17], yet clinic-based, “youth-friendly” approaches struggle to reach young people and up to 50% seek contraception outside of formal clinic facilities, primarily at community pharmacies [18].

Kenyan AGYW seeking contraception at community pharmacies may be a particularly marginalized demographic and behavioral subgroup: compared with those seeking contraception at public sector clinics, pharmacy-seekers are more likely to purchase emergency contraception (EC), report exchange sex, have multiple sexual partners, and report frequent EC use [19]. These data suggest that AGYW seeking contraception in pharmacies should be prioritized for tailored SRH programs to meet their unique needs.

Community pharmacies are a primary and preferred source of contraceptives for AGYW, but quality of care is insufficient. In studies from low- and middle-income countries (LMICs) in the WHO Africa region, including Kenya, AGYW cite convenience, privacy, and non-judgmental staff as reasons for preferring pharmacies [20–23]. However, pharmacy-based services are often limited in the range of contraceptive methods provided, and pharmacy staff lack requisite training and time to provide contraceptive counseling [18,24,25]. Recognizing that pharmacies outnumber health facilities and represent low-barrier entry points for youth at risk of undesired pregnancy, the WHO Global Accelerated Action for the Health of Adolescents highlights the need for enhanced contraceptive counseling in the pharmacy setting [26].

Person-centered contraceptive decision support tailored for AGYW in pharmacies using digital health technology could help address quality concerns and expand counseling reach. Choice of contraceptive method is a preference-sensitive decision [27], meaning that the best option is highly dependent on an individual’s values and preferences. Person-centered [28] contraceptive counseling that includes shared decision-making is the gold standard in contraceptive care and has been shown to enhance contraceptive satisfaction and continuation, promote equity, and protect against coercion [29–31]. Decision support tools are a valuable strategy to facilitate person-centered contraceptive counseling, as they can provide individualized information about options and side effects, address gaps and inaccuracies in knowledge, help identify preference-aligned choices and allow for higher quality and more time efficient client-provider communication [32]. A recent systematic review concluded that interactive, technology-based decision support tools have positive effects on contraceptive use, continuation, knowledge, and self-efficacy [33]. As connectivity expands in LMICs, digital health technologies are widely promoted as approaches to enhance quality and reach of health education and behavior change interventions in the context of severe provider shortage and task-shifting [34–36], but research on client-facing decision support tools is sparse [37,38]. To date, no contraceptive decision support tools tailored for AGYW, or for use in the pharmacy or other community-based setting, have been rigorously studied in LMICs or high-income regions. Defining effective and feasible strategies to enable AGYW access to quality contraceptive counseling in community-based delivery models is an urgent research priority.

Using a human-centered design framework, we partnered with AGYW and pharmacy providers to co-design the Mara Divas app: a digital health contraceptive decision support application tailored for AGYW in community pharmacy settings in Kenya. In this paper, we detail our iterative, participatory co-design process, specifying how stakeholder perspectives influenced the app’s design and content to prioritize person-centeredness, user-centeredness, and implementability.

## Materials and methods

### Setting

This study took place in Kisumu County, Kenya, a major population center in western Kenya that includes the city of Kisumu and its surrounding peri-urban and rural areas. Kisumu County is a multiethnic, multilingual region reflecting Kenya’s young demographics, with the majority of the population under 24 years of age [39]. Key SRH issues facing AGYW in the region include disproportionate rates of HIV and other STI acquisition, myriad barriers affecting access to pregnancy prevention, and unintended pregnancy [40,41]. According to the 2022 Demographic and Health Survey, the median age at first birth in Kisumu County is 19 years (national median is 21 years), and the modern contraceptive prevalence rate among sexually active unmarried women aged 15–19 is 44% [42].

In Kisumu county, as in most regions of Kenya, there are numerous private-sector retail pharmacies providing a variety of contraceptive methods. Licensed pharmacies, which are formally integrated into the revised National Family Planning Guidelines as frontline providers [43], typically stock condoms, oral emergency contraception (EC), oral contraceptive pills, and injectable contraception. Implants are increasingly available at pharmacies with attached clinical spaces and services are being actively scaled up in line with the National Family Planning Guidelines [44]; intrauterine device services are not generally available in pharmacies.

### Design team

We applied human-centered design methods with the goal of co-creating the intervention alongside its intended beneficiaries. Human centered design is an approach to solving problems through deeply empathetic engagement with people’s needs and lives, followed by iterative brainstorming and prototyping a potential service or product to optimize it for real people. Our multidisciplinary research team included four Kenya-based researchers and research staff with expertise in human-centered design and participatory methods (SWG, DCO, MA, CA), a Kenya-based design consultant (NKC), and three United States-based researchers (EKH, LO, RA) with expertise in global health, contraceptive counseling and clinical care, and computer science. EKH led the wireframing (creation of visual blueprints of the app layout and screens) and overall project, SWG and NC led data collection and analysis, EAB provided ethics expertise and liaisons with the Kenyan ministry of health officials, and LO and RA led a technical team of an additional five computer science students who provided software development expertise (please see acknowledgements). Our team has included a structured reflexivity statement in Supporting Information (S1 Text).

### Research ethics

The Kenya Medical Research Institute (KEMRI) Scientific Ethics Research Unit (KEMRI/SERU/CMR/4917) and the University of Washington Human Subjects Division (00020507) granted ethical approval for this study. Written informed consent (age 18 years or older) or assent (age 15–17 years) was obtained from all participants. A waiver of parental permission for minors was also approved; thus, adolescents aged 15–17 years were able to participate after a rigorous assent process without engaging their parents or guardians. A waiver of parental permission for minors to participate in research can be granted in Kenya under specific, regulated circumstances, which are outlined in the Guidelines for Conducting Adolescent HIV Sexual and Reproductive Health Research in Kenya [44]. Research procedures must present minimal risk to minors and the waiver must not negatively affect their rights and welfare. In this case, seeking parental permission for the current study could potentially put adolescent participants at risk for social harm due to loss of privacy around sexual activity and reproductive health choices. Our team employed a variety of approaches to safeguard vulnerable AGYW, including study staff training specific to protecting AGYW privacy and wellbeing, encouraging minors to seek the support or counsel of a trusted adult prior to participation (this is part of the assent form/process and is in line with the Guidelines), and individualized assent/consent discussions emphasizing voluntariness of participation and the limits of confidentiality assurance in a group setting.

This study was also approved by the Kenya National Commission for Science, Technology, and Innovation (NACOSTI, research permit P/24/33897) and the Kisumu County Health Management Team authorized the conduct of the study in Kisumu County.

### Design process

**Fig 1:**
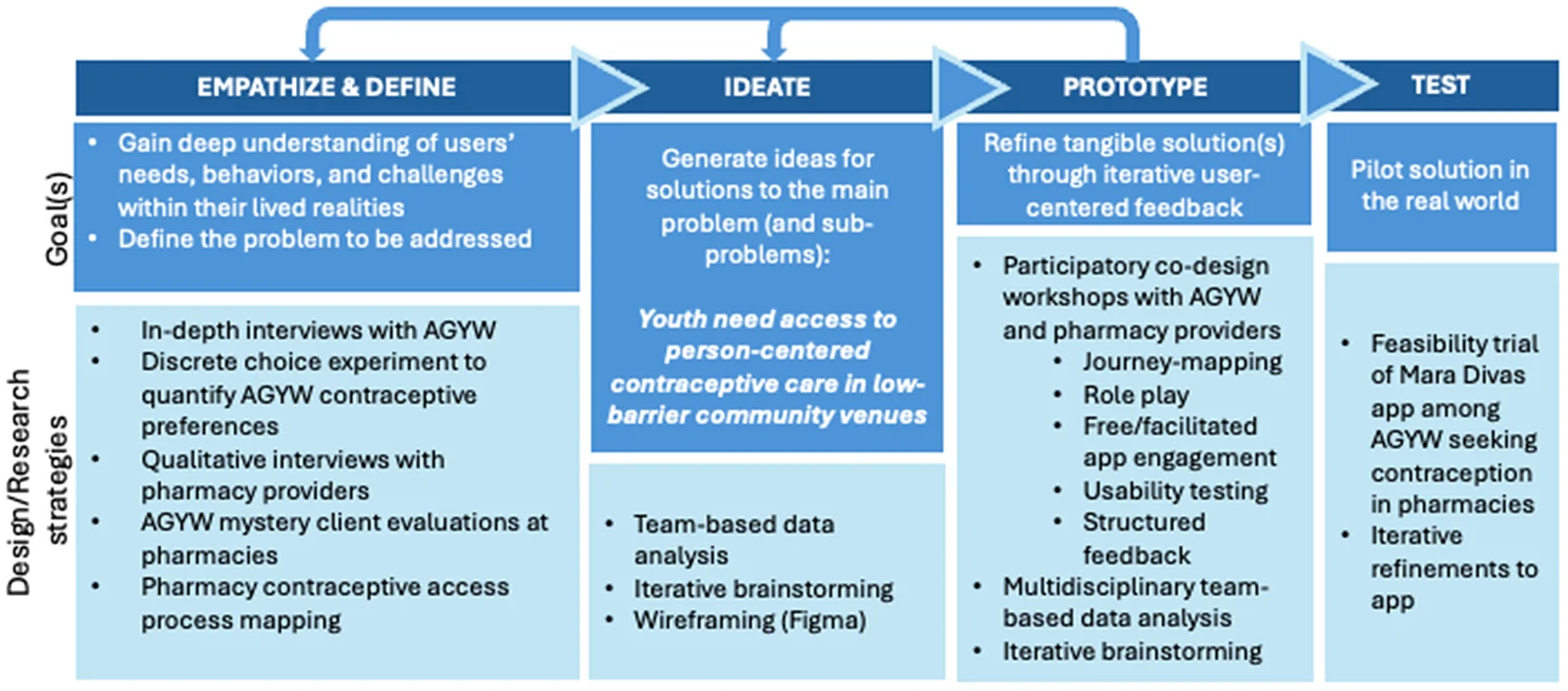
Goals of and research strategies used in the human-centered design process. Caption: AGYW: adolescent girls and young women.

In Fig 1, we summarize the phases of human-centered design, and the methods and strategies applied in each phase. This paper reports specifically on the ideation and prototyping phases; formative work and the feasibility trial are reported elsewhere.

#### Empathize and define.

Using a variety of qualitative and mixed methods approaches over several years, we sought to deeply understand AGYW’s contraceptive needs and preferences within their communities and lives [5,45,46], how reproductive health care-seeking and behaviors are influenced by sexual and reproductive empowerment [47,48], and quality of pharmacy-based contraceptive care for youth [49]. We developed a problem statement: *AGYW need access to person-centered contraceptive counseling in low-barrier community-based settings.*

#### Ideate.

**Fig 2:**
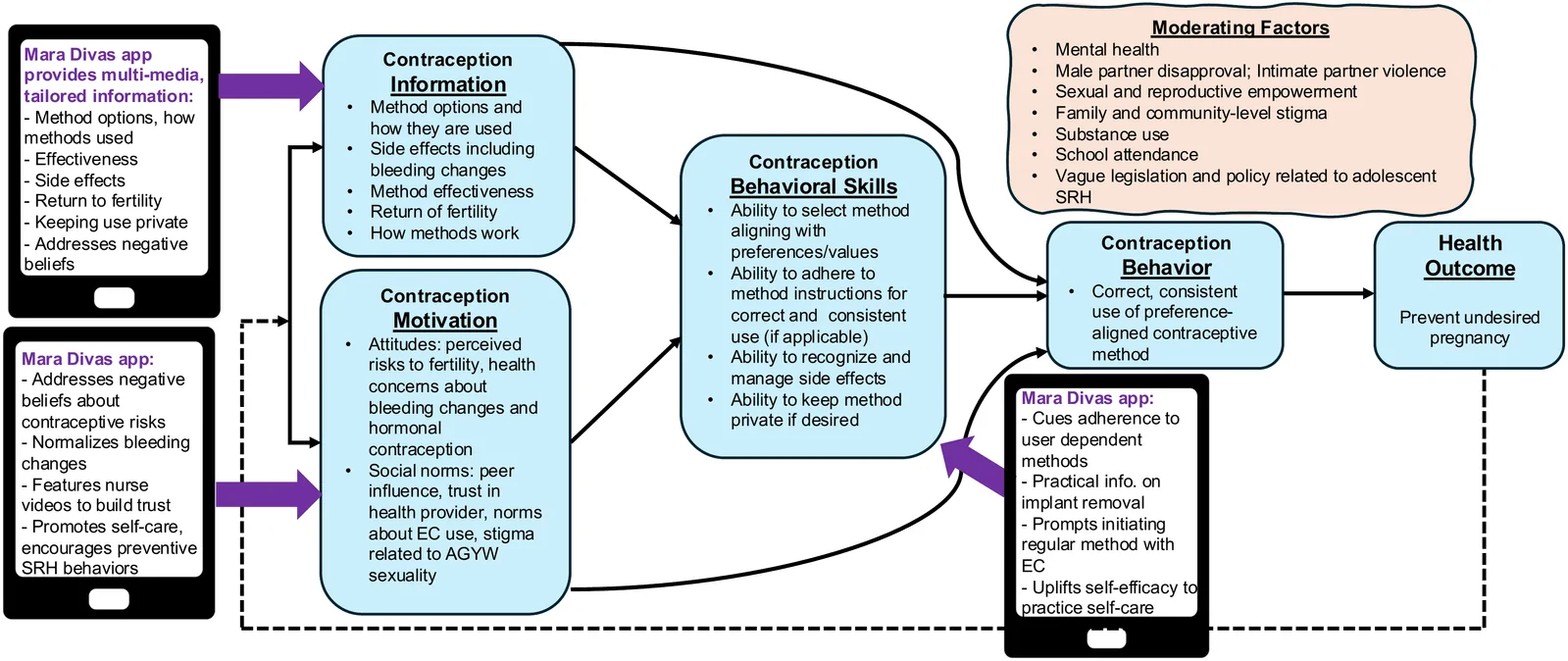
Information Motivation Behavioral Skills Model applied to AGYW desired contraceptive use. Legend: The Information-Motivation-Behavioral Skills (IMB) model highlights three core constructs that influence behavior change: information (accurate/comprehensive knowledge), motivation (personal attitudes and perceived social support), and behavioral skills (ability to perform related tasks and self-efficacy for those tasks). Adapted from Fisher *et al. Health Psychol* 2006, 25(4). AGYW: Adolescent girls and young women. EC: Emergency contraception. SRH: Sexual and reproductive health.

We brainstormed potential “solutions,” using the SCAMPER checklist [50] to prompt new perspectives with the goal of reaching marginalized youth—such as those with lower income and educational status, limited health literacy and access to information, and who reside in informal settlements. We engaged the question: *How might we support AGYW who desire pregnancy prevention to access contraceptive information and methods aligning with their values and preferences in the pharmacy setting?* Inspired by the *My Birth Control* web-based application (app) [29,51], we iterated on the idea of a digital health tool available in community pharmacies to provide a person-centered scaffolding for contraceptive information and decision-making, eventually choosing to design a tablet-based app due to prior research indicating that nearly 46% of AGYW representing our priority population did not have access to a private phone, let alone a smartphone with the ability to download an app or use the internet [45]. Intervention development was guided by the International Patient Decision Aid Standards best practices [52] for client-facing decision support tools and drew on the Information-Motivation-Behavioral skills (IMB) model. The IMB model was developed to understand and promote HIV prevention and treatment behaviors [53] and has been applied to many behavioral outcomes in sexual and reproductive health [54,55]. We brainstormed how the app content could target each IMB construct as outlined in Fig 2). Initial pen-and-paper sketches were translated into wireframes in Figma (Version 25.41.0, Figma, Inc., 2025), and computer science collaborators supervised software development by a technical team. The design team developed audio and visual content for the app, using stories from AGYW to create videos with AGYW actors and volunteer nurses in Kisumu to highlight AGYW experiences and priorities. A functional prototype app was built for the Android platform, targeting a tablet with a 10.5 inch display for ease of interaction. Future adaptability for other contexts with different linguistic or content needs was considered from the outset. For example, text content is housed in an online spreadsheet with a tab for each language; this content could be translated into other languages and relatively easily switched out by altering the code to link a new spreadsheet. Audio and video could be re-recorded in other languages and cultural contexts to represent local communities.

### App overview

The app is designed for use with headphones on a tablet that is physically located in the pharmacy. An appropriate pharmacy has private space where AGYW can engage with the app without being observed or heard by other pharmacy clients or passersby. It is common in Kenya for pharmacies to provide counseling and injections to clients in private consultation rooms. When AGYW request contraceptive methods or information, providers would offer access to the tablet-based app prior to purchase. After launching the app, users see a start page that frames contraceptive choice as preference-sensitive and encourages AGYW to be informed. The home page has six main sections, which were inspired by the results of a discrete choice experiment to understand AGYW priorities [45]: *What are my options?, What will happen to my period?, How long does the method work?, What is my chance of getting pregnant?, Can I keep it private?,* and *What if I’m ready to have a baby?* The options section includes methods that are widely available in Kenya: external and internal condoms, oral contraceptive pills, oral EC, injectables, copper intrauterine device (IUD), and implants. Each section has text offered in three languages (English, Kiswahili, and Dholuo) with audio available, videos of peer stories and provider explanations, and options to “learn more” about other SRH topics, preparing for a healthy pregnancy, mechanisms of action for contraceptive methods, etc. After reviewing at least one section, AGYW can take a quiz to help highlight how their specific preferences may align with contraceptive methods. Users can then purchase their desired method or receive a referral to a nearby health facility if the chosen method is not available at the pharmacy. The Mara Divas app may be, but is not required to be, used in collaboration with a pharmacy provider, differentiating it from other tools in which the app output is used to promote shared decision-making with a health provider [51,56].

#### Prototype.

We conducted full-day participatory workshops in Kisumu to co-design and refine the prototype among AGYW and pharmacy providers. Community-based participant recruitment took place between May-July d. Eligible AGYW were age 15–24 years, reported purchasing contraception in a community pharmacy in the last 3 months, and not currently pregnant. Workshops employed methods such as journey mapping and role play to understand key pain points of accessing contraception in a pharmacy and how the app could fit into the journey of before, during, and after the pharmacy interaction. Workshops also used group interviewing to understand experiences during individual and guided engagement with the app prototype and a structured feedback-generating activity called “I like, I wish, What if” [57] in which users reflected on different features of the app. Facilitators conducted usability testing in which participants were assigned different “tasks” to perform in the app and were encouraged to “think out loud” as they completed them [58]. Workshop activities were audio-recorded and the relevant portions were transcribed. Visual workshop artifacts (See S1 Fig), including groups of sticky notes, were transcribed into electronic sticky notes on Mural, an online visual work platform. The design team met after and between workshops to collaborate on Mural, iteratively analyzing workshop data to generate insights. Data sources included photos, artifacts, official written notes, and transcripts. The technical team iteratively modified the prototype in response to insights. Throughout prototyping, we considered how our design influenced person-centeredness, acceptability (satisfaction with various aspects of the innovation) [59], appropriateness of the app for the pharmacy setting (relevance, suitability, usefulness) [59] among users and providers, and feasibility of implementation in the real world.

#### Test.

We conducted a feasibility trial of the app at six community pharmacies in Kisumu County; these findings will be reported elsewhere.

## Results

We conducted a total of four co-design workshops: three among AGYW (Workshops 1–3) and one among pharmacy providers. The AGYW workshops engaged 10–12 participants each (n = 33), with a median age of 19 years (IQR 17–21); workshops were stratified by age, with workshop 2 focused on 15–17 year-olds. The median ages of participants in workshops 1–3 were 20, 17, and 21, respectively. Nearly half (49%) were students in secondary school or vocational colleges, most (85%) lived with a parent, and 73% owned a mobile phone (4/12 or 33% in workshop 2). Half (17/33, 52%) of AGYW participants reported access to a smartphone. Most (58%) had ever given birth. Most used contraceptive methods were condoms (76%), EC (39%), and implants (39%). Among pharmacy providers (n = 10), 6 were female, and most were pharmacy technicians with over 5 years of experience.

### Insights and prototype refinements

**Fig 3:**
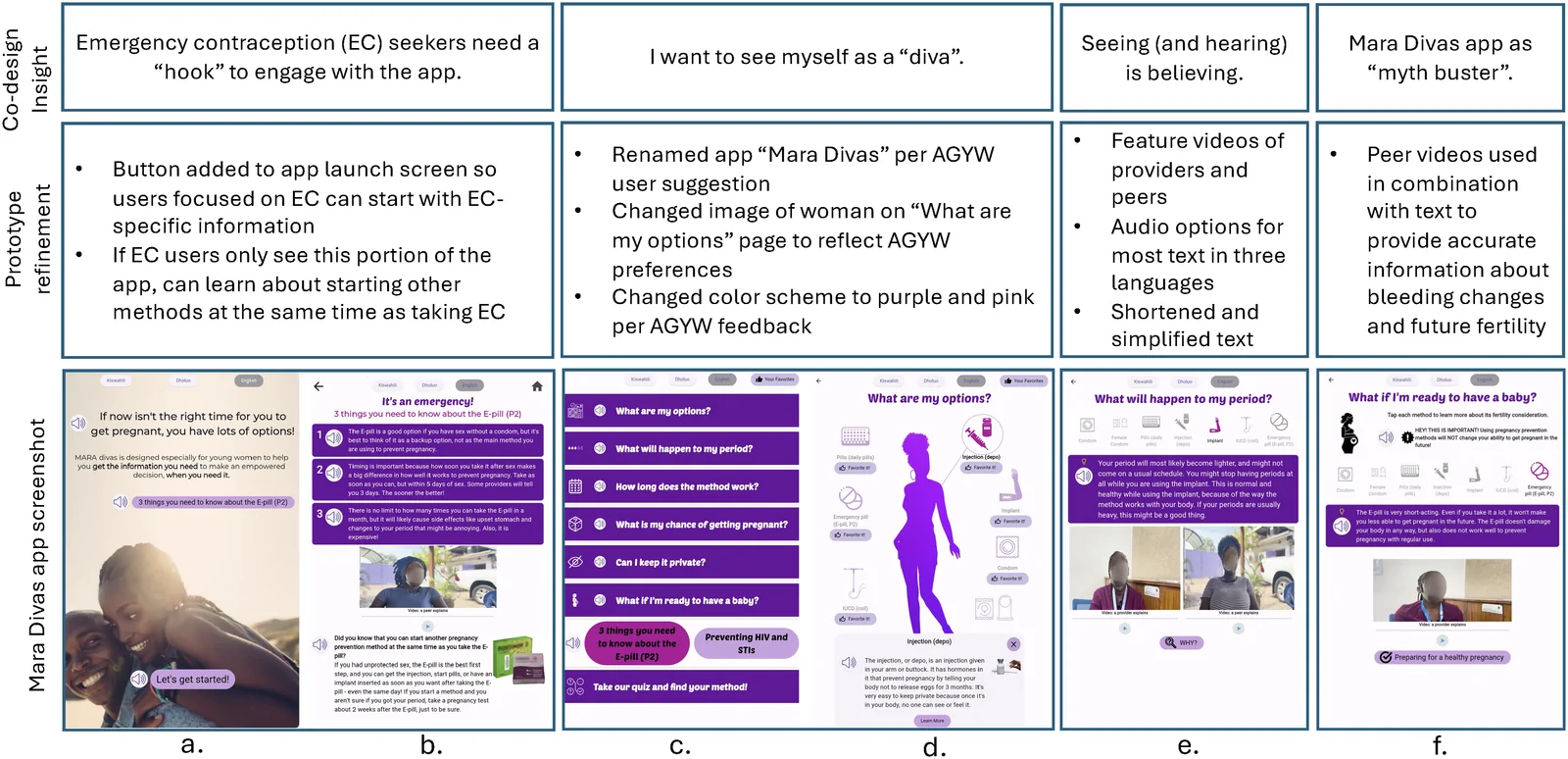
Selected co-design insights and prototype refinements. Legend: AGYW: adolescent girls and young women.

The co-design process yielded multiple design insights relevant to prototype design and implementability; selected insights’ influence on the final Mara Divas app prototype and example screenshots are shown in Fig. 3a-f, and illustrative quotations are summarized in Table 1.

**Table 1:** Themes and illustrative quotations from the co-design process.

	Theme (*Co-design activity)*	*Quotation* (Workshop ID)
a	AGYW sensitivity to provider facial expression, tone of voice, perceived judgment (*Journey mapping)*	*Okay you know what, maybe he [pharmacy provider] has that harsh look on his face, or not that they are judging you but the way they are looking at you and then once you get to him maybe I wanted to tell you something then I will be like, “where will I start from?” So it will force me to hide.* (**AGYW,** Workshop 2)
b	AGYW scan the pharmacy environment to assess whether conditions allow for contraceptive access (*Journey mapping)*	*Actually you should think because let me say you have gone for that implant, obvious they will not put for you that implant there in front, because another person might come and they find you let me say for example he was your neighbor and they have come and they find you are being inserted implant in front there. So you have to think or you do this, when you are going to the pharmacy, you are going to get painkillers, and sometimes it is the emergency pill, just go to that pharmacy and try and figure out how the frame of the pharmacy is, yeah.* (**AGYW**, Workshop 1)
c	Many AGYW avoid provider counseling and seek EC and other contraceptive methods at “rocket speed” *(Journey mapping)*	*There are a few [AGYW] who will come and say, like, “I used it [EC] last month, is it safe to use it again?” Very few will ask you. Or “I’m on my periods, can I use it tomorrow?” These are very few. But majority are the ones in a hurry as they have said, “give me P2” [EC]. Some will even send boda boda [motorcycle taxi] guys. They will send their younger siblings.* (**Provider**)
d	A subset of AGYW would prefer to have the app available via smartphone prior to going to the pharmacy *(Journey mapping)*	*I feel like the entire app should be in the app store because, like, most of the people fear going to the pharmacy… A lot of people fear approaching those people [providers] in the pharmacy. So at least you go there when you have all the information. And this app has all the information… So I feel that if you have the app it would be better if we share with our friends so that we can minimize or reduce the myths in the community.* (**AGYW**, Workshop 1)
e	Provider beliefs/biases about contraceptive risk to future fertility conflict with the app content *(Journey mapping, App engagement and usability testing)*	*Some people might even fail to return to fertility…I tell you “fine, you’re going to enjoy sex but remember one day you’ll get a very handsome man, a very caring husband, but the Lord will not give you that child that you want”…Some of these family planning methods can even interfere with you until you get fibroids…You end up going to theater until you get your fibroids and they even remove those, your ovaries.* (**Provider**)
f	Videos were helpful in addressing inaccurate contraceptive beliefs *(App engagement and usability testing)*	*I think...It has the videos…the provider experiences is explaining more about the information that is there. And the woman [peer] giving the experience on what the doctor told her and what she was told to do and how she followed his instruction, how she used it and what she experienced. It [the video] also gives you hope, despite the myths about contraceptives.* (**AGYW**, Workshop 2)
g	App makes it easier to find and understand contraceptive options *(App engagement and usability testing)*	*If you go to Google, it has a lot of options and it’s confusing. But this app, it has specified what you want. I think it has been written in simple terms, not the heavy, heavy English…people struggle to understand.* (**AGYW**, Workshop 3)
h	App addresses inaccurate beliefs *(App engagement and usability testing)*	*Me I knew if you put family planning let’s say implant for 5 years it is just supposed to wait after 5 years is when you can remove it but I have seen here they have written you can even remove it before 5 years.* (**AGYW**, Workshop 2)

Caption: AGYW: adolescent girls and young women; EC: emergency contraception

### Journey mapping

#### Managing anxiety: Demand for “rocket speed” service.

Among AGYW participants, the experience of purchasing contraception in a pharmacy was consistently portrayed as fraught with anxiety, related both to intense fear of pregnancy coexistent with fear of judgment by pharmacy providers, partners, and family members. Their ultimate goal was relief from these sources of anxiety, for example by leaving the pharmacy with a contraceptive solution that would not be discovered by others. Privacy and confidentiality were more important than any other aspect of the experience, surpassing provider expertise and available contraceptive options. AGYW described constantly scanning the environment to determine whether it was safe to open up to a provider about needing contraception, or whether they would just ask for painkillers at the last minute or leave (Table 1a). They might walk around the pharmacy to investigate or perhaps wear a head covering to disguise their identities. Their vigilance applied to factors such as provider gender, age, facial expressions, tone of voice; distance of the pharmacy from home or school; and the presence of other clients who could overhear or judge. Most AGYW, frequently consulting peers or family members, described trying to make a decision about which method they would purchase ahead of time to minimize the potential of being seen at the pharmacy, but expressed a simultaneous desire for comprehensive contraceptive information that included choosing the best method for their needs and covered side effects. Often, they struggled to reconcile the absence of ideal conditions and their immediate need for pregnancy prevention, and settled for the most rapid, discreet interaction possible (Table 1b). In parallel, pharmacy providers spoke extensively of AGYW clients’ demand for “rocket speed” services, noting both the “shyness” (using coded language, sending children in their place, asking for pills to be removed from packaging) and the “assertiveness” of AGYW who will seek services elsewhere if they feel threatened by providers’ questions – even when providers are trying to provide contraceptive counseling for the clients’ benefit (Table 1c).

#### Integrating the app into the journey: Fit and tensions.

When journey maps were revisited after engaging with the app prototype, AGYW integrated the app into the journey immediately after greetings were exchanged with the provider, allowing them to bypass being “grilled” by the provider and explore their contraceptive options without fear of judgment in a private consultation room. That said, users often had questions that arose during app engagement as they were exposed to new contraceptive information, and wanted the opportunity to consult with pharmacy providers after app engagement. AGYW participants in two workshops also voiced a demand for access to the app via mobile phone, so they could use it before and after presenting to the pharmacy, could spend more time with it, involve friends and male partners in decision-making, and review side effects and other information after starting a new method (Table 1d). Providers emphasized the necessity of balancing empathy for AGYW with their own “self-preservation,” referring to the need to protect their income generation and reputation. Providers generally felt a sense of responsibility to help young women navigate pregnancy prevention, and felt the app could save them time by supporting contraceptive knowledge and making their jobs easier. Tensions also emerged, as some providers expressed strong biases about the safety of AGYW using certain methods, especially repeated doses of EC and use of injectable contraception, their views at times conflicting with app content (Table 1e). Some had concerns about enabling “promiscuous” sexual behavior: as one provider stated, *“you help them with their mischiefs”*. Providers also worried about regulatory bodies and community members accusing them of failing to get parental consent for minors’ contraceptive use, wondering if providing access to the Mara Divas app would call unwanted attention to what pharmacies were already doing—contraception provision to AGYW.

Providers introduced a key insight into their journey map after providing feedback on app structure and content: AGYW seeking EC need a “hook” to motivate engagement with the app, as they are less likely to be interested in understanding all their contraceptive options when they perceive only a single option (oral EC) after unprotected sex. Providers and AGYW described the experience of seeking EC at the pharmacy as representing the pinnacle of “rocket speed” interactions. These insights inspired a prototype refinement, adding a button on the app launch screen (Fig 3a, 3b) to attract EC seekers: “3 things you need to know about the E-pill”. This button was also added to the home screen (Fig 3c).

### App engagement and usability testing

#### Literacy and navigability: Seeing (or hearing) is believing.

AGYW preferred the video and visual content to text, though audio narration of text was popular. They particularly gravitated toward the videos of peers explaining their experience with common bleeding changes associated with contraceptive use, privacy, and fertility concerns (Table 1f). Each screen (including video/audio) could be engaged in Kiswahili, Dholuo, or English; AGYW switched languages within and between screens frequently during app engagement (Table 1g). In usability testing, the 15–17 year-old workshop group, who had minimal experience with smartphones or use of social media and other apps, had difficulty with tasks such as finding method-specific information or navigating back to the home screen. We added additional instructions, colored buttons, larger icons, and scrollbars to the prototype to enhance navigability, as well as generally shortening and simplifying text to tailor for limited-literacy AGYW with less contraceptive experience or knowledge.

#### Reflection of a “diva”.

AGYW participants made a variety of suggestions about the color scheme and visual attractiveness of the app, desiring the app to project a confident, powerful, sexy persona summarized by one workshop group as a “diva”—an aspirational icon of who they wanted to be or become. They had strong opinions about how the woman on the “What are my options?” page should be “shaped” and what she should wear, prompting a variety of mock-ups with different colors and figures. While the word *mara* means “mine,” or “my own” in the Luo language, users did not initially see the connection to contraceptive autonomy and choice and preferred the word “diva” to be included in the app name.

#### Mara Divas app as “myth buster”.

The app content was designed to provide accurate contraceptive information about available methods using digital mixed media, and to specifically address common beliefs about contraceptive harms that are not evidence-based. When asked about if the app could change their pre-existing ideas about contraception, AGYW users generally described feeling confident that the app content was a source of information they could trust—potentially more than pharmacy providers in some cases. In two of three AGYW workshops, the term “myth buster” was generated in feedback sessions, and participants frequently reflected on the videos of provider and AGYW stories as compelling in changing their thinking about the benefits and risks of various methods. As expected, bleeding changes and future fertility were top of mind for users. Several explained how using the app influenced their perspectives on methods; for example, several AGYW explained how they had previously thought that intrauterine devices rust when exposed to sperm and that’s why they are unsafe for young unmarried women who are having frequent sex, and others had previously believed that injectable contraception could destroy the ovaries or using EC could cause birth defects. The app’s content also addressed the belief that one could not have a contraceptive implant removed prior to its longest duration of use (Table 1h).

#### Give me fewer options?.

The quiz at the end of the app was more controversial than other app features, with the general critique that it was overwhelming or that the recommendations after going through the quiz were not directive enough. Rather than finding it fun or interactive, some felt it was like “taking an exam” (quote from sticky note, workshop 3) to read all the questions and was too time consuming. This critique was strongest in the workshop with the youngest participants. In parallel with other app refinements, the content was simplified as much as possible and amount of text was reduced. Given that the quiz was designed to help users synthesize preference-aligned options—not to tell users which method they should use—its value was uncertain after the prototyping phase.

## Discussion

### Principal findings

In this study, we leveraged human-centered design approaches to iteratively co-design a novel pharmacy-based contraceptive decision support app tailored for youth. Our findings offer key insights into optimizing the app’s acceptability and appropriateness for adolescent girls and young women, as well as how a digital health contraceptive decision support tool can be integrated into youth contraceptive care journeys in the pharmacy setting in meaningful and feasible ways. Our experience engaging youth in participatory workshops emphasizes the diversity of needs and self-efficacy among 15–24 year-olds: participants in the co-design process had highly variable digital literacy, reading comprehension, and contraceptive knowledge/experience. While younger, less tech-savvy youth, some of whom have no smartphone experience, and older AGYW who own smartphones may experience and use app-based contraceptive decision support very differently, they have similar needs for limited-literacy contraceptive information. AGYW with limited tech literacy may require enhanced orientation to the app by the pharmacist than others who are used to navigating apps, and may benefit from more than one “dose” of the app. The co-design process elucidated intervention features that influenced the acceptability and appropriateness of the app; many AGYW were drawn to the videos and audio text narration over reading text, and they frequently switched between languages by app screen, reflecting the norm of multilingualism in the region and use of blended English and Kiswahili (“Sheng”) slang among youth. However, there are significant trade-offs in prioritizing reach among the youngest, most socio-economically marginalized AGYW with a tablet-based app physically in the pharmacy. Given the anxiety and stigma that promote “rocket speed” interactions among AGYW seeking contraception in pharmacies, youth with access to a smartphone and internet connection advocated for the option to interact with the app in the “before pharmacy” part of the care journey as well as at the pharmacy, in part so they could share the content with peers and male partners. Additionally, when used outside the pharmacy space, the app may have more potential to address concerns about side effects after starting a new method (“after pharmacy”). Future research into a smartphone version of Mara Divas will need to balance privacy and equity concerns with reaching specific profiles of AGYW. The wide range of digital literacy among AGYW even in an urban center in Kenya—a lower-middle income country with extremely high digital connectivity [60]—underscores the importance of considering who would be excluded from digital health interventions due to limited access to a connected device. Based on literature review and experience [61,62], digital literacy is expected to be even more variable among youth in rural areas in Kenya and in lower-income countries.

A central design team question was whether an app would be “enough” to shift negative beliefs about contraception that are not clinically accurate but are pervasive in users’ communities, particularly about bleeding pattern safety and future fertility. While in alignment with the Stevens *et al.* commentary (2023) [63], recommending neutral language about negative contraceptive beliefs to avoid paternalistic dismissal of user concerns, we also recognize that such beliefs are a critical barrier to contraceptive agency among youth [5,64–66^]^ and few interventions have directly addressed negative beliefs [67]. Our data suggest that users found the app to be a trustworthy source of information and many users reported changing their minds about methods based on app content. Videos from peer and provider perspectives were especially effective in “myth busting” – using the words of participants. Repeated exposure to evidence-based information about contraceptive side effects and person-centered counseling around contraceptive choice may further empower contraceptive choice, as might using resources like Mara Divas in peer groups and/or with male partners. Ongoing pilot testing in real-world pharmacies and eventual evaluation of clinical effectiveness will further elucidate the utility of the app in supporting preference-aligned contraceptive decisions in the pharmacy setting.

### Clinical and research implications

The inherent challenges to providing a higher quality of contraceptive care in community pharmacies in LMICs are well-documented, including competing provider priorities in the private sector, limited provider training, privacy challenges, client preferences, and counterfeit product concerns [20,49,68]. Notwithstanding, our findings highlight the promise of digital health contraceptive decision support to reach marginalized youth who most need person-centered contraceptive care. Insights from AGYW and pharmacy provider workshops were consistent with prior literature calling attention to limited contraceptive knowledge among youth as a key hurdle to desired contraceptive use [2,65]. Drawing on the IMB model, information is a necessary element of behavior change related to preference-aligned contraceptive use—but insufficient without motivation and behavioral skills.

Our findings contribute to understanding the critical role of emergency contraception among AGYW who access contraception at pharmacies. Reflecting insights from the journey mapping process, youth EC seekers are strongly motivated to prevent pregnancy but less motivated to participate in contraceptive counseling due to anxiety and perceived lack of options. Pharmacy-based interventions must present EC-users with information that is immediately relevant in order to engage them, such as how to optimize EC’s effectiveness, how it works, and how to prevent pregnancy after EC use—including other methods that can be initiated at the same time.

The reality that some AGYW desired more directive recommendations from the app is relatable in light of their limited exposure to detailed information about contraceptive options: the amount of information, packaged to elevate individual preferences and needs rather than specific methods, was overwhelming to some. Yet, directive recommendations are not in line with the framework of person-centered care and is unlikely to advance preference-aligned contraceptive care. Research with additional users is needed to understand how to best help users synthesize information that is most relevant to their choice of method; co-design of a replacement to the quiz may be needed. Strategies to overcome person-centered information overload could include repeated exposure to the app in the pharmacy, making the app accessible on youth devices, creation of “beginner” and “advanced” tracks within the app. Use of artificial intelligence to answer specific questions or tailor user experience could also help balance person-centeredness and comprehension with appropriate safeguards. Furthermore, pharmacy providers remain key players in the provision of person-centered contraceptive care in pharmacies, with interventions like Mara Divas aiming to support and improve quality of care. The co-design process reiterated the need for provider training, specifically related to negative beliefs about contraception among youth, judgmental attitudes or bias towards AGYW, and re-orientation towards harm reduction that meets some AGYW’s needs rather than a one-size-fits-all intervention.

### Strengths and limitations

Strengths of this study include its participatory, community-engaged data collection and analytical methods, drawing on the experience of a multidisciplinary team with dedicated human-centered design and content expertise. Our human-centered design approach allowed for the co-design of the app user experience with its intended users: adolescent girls and young women who seek contraceptive care at pharmacies. We anticipate that iterative refinements centered on youth input will ultimately yield enhanced appropriateness and acceptability among youth, and overall feasibility of implementation of the intervention in real-world pharmacies. This research also has important limitations. We engaged youth in generally low-income urban and peri-urban communities in a single Kenyan county, and our findings are not intended to be directly generalized to diverse groups of AGYW in other geographic areas. Youth who seek or want to access contraception in pharmacies in rural areas are specifically not represented in this study. Due to logistical constraints, we were unable to include a youth representative or pharmacy provider on the design team during initial app ideation, and recognize that inclusion during this phase may have been even more generative. However, human-centered design is not a linear process, with AGYW input used to iterate on insights and content created throughout the app’s design and extensive qualitative research was conducted during earlier phases (emphathize and define). While this app is intentionally focused on reaching adolescent girls and young women, our findings highlight the need to include male partners in ongoing work with diverse stakeholders to further enhance its person-centeredness.

## Conclusions

Community pharmacies are the preferred source of contraceptive methods for a large proportion of AGYW in Kenya and other LMICs and remain a relatively untapped opportunity to improve the reach, quality, and person-centeredness of contraceptive care for youth. We used human-centered design approaches to develop and prototype the first contraceptive decision support app tailored for AGYW in the pharmacy setting, allowing for co-creation of the app content and user experience to optimize acceptability, appropriateness, and implementability. While the Mara Divas app is highly tailored for a specific age group and region of Kenya using extensive formative research, our team’s technical approach demonstrates how future adaptability to diverse contexts and scalability can be considered at the earliest phases of digital health intervention design. In other words, it would be technically simple to switch out text, audio, and video content with content tailored for a new setting. During the next phase of this project, we will conduct additional stakeholder analysis and a feasibility trial to pilot the app in Kisumu County pharmacies and evaluate preliminary effectiveness alongside implementation determinants and outcomes.

## Supporting information

S1 Text Structured reflexivity statement. (PDF)

S1 Fig Journey mapping artifacts. Legend: Clockwise from top left: Participants brainstorm during journey mapping; example of journey map artifact; example of journey mapping product among pharmacy providers; workshop role play.
(TIFF)

S1 File Inclusivity in Global Research. (DOCX)

## References

[1] United Nations. Transforming our World: The 2030 Agenda for Sustainable Development. 2015 [cited 2023 Aug 10]. Available from: https://www.un.org/sustainabledevelopment/

[2] Yakubu I, Salisu WJ. Determinants of adolescent pregnancy in sub-Saharan Africa: a systematic review. Reprod Health. 2018;15(1):15. doi: 10.1186/s12978-018-0460-4 29374479 PMC5787272

[3] Mathur S, Okal J, Musheke M, Pilgrim N, Kishor Patel S, Bhattacharya R, et al. High rates of sexual violence by both intimate and non-intimate partners experienced by adolescent girls and young women in Kenya and Zambia: Findings around violence and other negative health outcomes. PLoS One. 2018;13(9):e0203929. doi: 10.1371/journal.pone.0203929 30212561 PMC6136792

[4] Onono MA, Brindis CD, White JS, Goosby E, Okoro DO, Bukusi EA, et al. Challenges to generating political prioritization for adolescent sexual and reproductive health in Kenya: A qualitative study. PLoS One. 2019;14(12):e0226426. doi: 10.1371/journal.pone.0226426 31856245 PMC6922405

[5] Harrington EK, Casmir E, Kithao P, Kinuthia J, John-Stewart G, Drake AL, et al. “Spoiled” girls: Understanding social influences on adolescent contraceptive decision-making in Kenya. PLoS One. 2021;16(8):e0255954. doi: 10.1371/journal.pone.0255954 34383836 PMC8360567

[6] Embleton L, Braitstein P, Di Ruggiero E, Oduor C, Wado YD. Sexual and reproductive health service utilization among adolescent girls in Kenya: A cross-sectional analysis. PLOS Glob Public Health. 2023;3(2):e0001508. doi: 10.1371/journal.pgph.0001508 36963079 PMC10021741

[7] Sully EA, Biddlecom A, Darroch JE, Riley T, Ashford LS, Lince-Deroche N. Adding it up: Investing in Sexual and Reproductive Health 2019. 2020. https://www.guttmacher.org/sites/default/files/report_pdf/adding-it-up-investing-in-sexual-reproductive-health-2019.pdf

[8] Riley T. Fact sheet. Adding it up: investing in contraception and maternal and newborn health for adolescents in Kenya. Guttmacher Institute. 2019. https://www.guttmacher.org/fact-sheet/adding-it-up-contraception-mnh-adolescents-kenya Accessed 2020 January 28.

[9] Ganchimeg T, Ota E, Morisaki N, Laopaiboon M, Lumbiganon P, Zhang J, et al. Pregnancy and childbirth outcomes among adolescent mothers: a World Health Organization multicountry study. BJOG. 2014;121(Suppl 1):40–8. doi: 10.1111/1471-0528.12630 24641534

[10] World Health Organization. Global health estimates 2015: deaths by cause, age, sex, by country and by region, 2000-2015. Geneva. 2016. http://www.who.int/healthinfo/global_burden_disease/estimates/en/index1.html

[11] Ziraba AK, Izugbara C, Levandowski BA, Gebreselassie H, Mutua M, Mohamed SF, et al. Unsafe abortion in Kenya: a cross-sectional study of abortion complication severity and associated factors. BMC Pregnancy Childbirth. 2015;15:34. doi: 10.1186/s12884-015-0459-6 25884662 PMC4338617

[12] Chandra-Mouli V, Camacho AV, Michaud P-A. WHO guidelines on preventing early pregnancy and poor reproductive outcomes among adolescents in developing countries. J Adolesc Health. 2013;52(5):517–22. doi: 10.1016/j.jadohealth.2013.03.002 23608717

[13] Plesons M, H MP, Cole CB, H MP, Hainsworth G, Ed M. Forward, together: A collaborative path to comprehensive adolescent sexual and reproductive health and rights in our time. J Adolesc Health. 2019;65. doi: 10.1016/j.jadohealth.2019.09.009 31761004

[14] Håkansson M, Oguttu M, Gemzell-Danielsson K, Makenzius M. Human rights versus societal norms: A mixed methods study among healthcare providers on social stigma related to adolescent abortion and contraceptive use in Kisumu, Kenya. BMJ Global Health. 2018. doi: 10.1136/bmjgh-2017-000608 PMC5841529 29527357

[15] Miller LE, Zamudio-Haas S, Otieno B, Amboka S, Odeny D, Agot I. We don’t fear HIV. We just fear walking around pregnant.: A qualitative analysis of adolescent sexuality and pregnancy stigma in informal settlements in Kisumu, Kenya. 10.1111/sifp.12178 PMC8932675 34766351

[16] Fikree FF, Lane C, Simon C, Hainsworth G, MacDonald P. Making good on a call to expand method choice for young people - Turning rhetoric into reality for addressing Sustainable Development Goal Three. Reprod Health. 2017;14(1):53. doi: 10.1186/s12978-017-0313-6 28399923 PMC5387301

[17] Chandra-Mouli V, Akwara E. Improving access to and use of contraception by adolescents: What progress has been made, what lessons have been learnt, and what are the implications for action? Best Pract Res Clin Obstet Gynaecol. 2020;66:107–18. doi: 10.1016/j.bpobgyn.2020.04.003 32527659 PMC7438971

[18] Radovich E, Dennis ML, Wong KLM, Ali M, Lynch CA, Cleland J. Who meets the contraceptive needs of young women in sub-Saharan Africa? J Adolesc Health. 2018;62(3):273–80. doi: 10.1016/J.JADOHEALTH.2017.09.013 29249445

[19] Pintye J, Odoyo J, Nyerere B, Achieng P, Araka E, Omondi C, et al. Nurse-facilitated preexposure prophylaxis delivery for adolescent girls and young women seeking contraception at retail pharmacies in Kisumu, Kenya. AIDS. 2023;37(4):617–23. doi: 10.1097/QAD.0000000000003447 36653342 PMC9974532

[20] Gonsalves L, Hindin MJ. Pharmacy provision of sexual and reproductive health commodities to young people: a systematic literature review and synthesis of the evidence. Contraception. 2017;95(4):339–63. doi: 10.1016/j.contraception.2016.12.002 28025018

[21] Gonsalves L, Wyss K, Gichangi P, Hilber AM. Pharmacists as youth-friendly service providers: documenting condom and emergency contraception dispensing in Kenya. Int J Public Health. 2020;65(4):487–96. doi: 10.1007/s00038-020-01348-9 32472373 PMC7275003

[22] Gonsalves L, Wyss K, Cresswell JA, Waithaka M, Gichangi P, Martin Hilber A. Mixed-methods study on pharmacies as contraception providers to Kenyan young people: who uses them and why? BMJ Open. 2020;10(7):e034769. doi: 10.1136/BMJOPEN-2019-034769 32641322 PMC7348460

[23] Corroon M, Kebede E, Spektor G, Speizer I. Key Role of Drug Shops and Pharmacies for Family Planning in Urban Nigeria and Kenya. Glob Health Sci Pract. 2016;4(4):594–609. doi: 10.9745/GHSP-D-16-00197 28031299 PMC5199177

[24] Gonsalves L, Kamuyango A, Chandra-Mouli V. Pharmacies: an important source of contraception for some adolescents, but not a panacea for all. Sex Reprod Health Matters. 2023;31(1):2221883. doi: 10.1080/26410397.2023.2221883 37417799 PMC10332186

[25] Gonsalves L, Wyss K, Gichangi P, Say L, Martin Hilber A. Regulating pharmacists as contraception providers: A qualitative study from Coastal Kenya on injectable contraception provision to youth. PLoS One. 2019;14(12):e0226133. doi: 10.1371/journal.pone.0226133 31856196 PMC6922368

[26] Global accelerated action for the health of adolescents: guidance to support county implementation. Geneva: World Health Organization. 2023. https://www.who.int/publications/i/item/9789240081765

[27] Charles C, Gafni A, Whelan T. Shared decision-making in the medical encounter: what does it mean? (or it takes at least two to tango). Soc Sci Med. 1997;44(5):681–92. doi: 10.1016/s0277-9536(96)00221-3 9032835

[28] Institute of Medicine. Crossing the Quality Chasm: A New Health System for the 21st Century. Institute of Medicine. 2001.

[29] Dehlendorf C, Fitzpatrick J, Fox E, Holt K, Vittinghoff E, Reed R, et al. Cluster randomized trial of a patient-centered contraceptive decision support tool, My Birth Control. Am J Obstet Gynecol. 2019;220(6):565.e1–565.e12. doi: 10.1016/j.ajog.2019.02.015 30763545

[30] Dehlendorf C, Krajewski C, Borrero S. Contraceptive counseling: best practices to ensure quality communication and enable effective contraceptive use. Clin Obstet Gynecol. 2014;57(4):659–73. doi: 10.1097/GRF.0000000000000059 25264697 PMC4216627

[31] Dehlendorf C, Grumbach K, Schmittdiel JA, Steinauer J. Shared decision making in contraceptive counseling. Contraception. 2017;95(5):452–5. doi: 10.1016/j.contraception.2016.12.010 28069491 PMC5466847

[32] Stacey D, Lewis KB, Smith M, Carley M, Volk R, Douglas EE, et al. Decision aids for people facing health treatment or screening decisions. Cochrane Database Syst Rev. 2024;1(1):CD001431. doi: 10.1002/14651858.CD001431.pub6 38284415 PMC10823577

[33] Goueth RC, Maki KG, Babatunde A, Eden KB, Darney BG. Effects of technology-based contraceptive decision aids: a systematic review and meta-analysis. Am J Obstet Gynecol. 2022;227(5):705. doi: 10.1016/J.AJOG.2022.06.050 35779590 PMC9800645

[34] L’Engle KL, Mangone ER, Parcesepe AM, Agarwal S, Ippoliti NB. Mobile Phone Interventions for Adolescent Sexual and Reproductive Health: A Systematic Review. Pediatrics. 2016;138(3):e20160884. doi: 10.1542/peds.2016-0884 27553221

[35] WHO guideline: recommendations on digital interventions for health system strengthening. Geneva: World Health Organization. 2019. https://iris.who.int/bitstream/handle/10665/311941/9789241550505-eng.pdf?sequence=31 31162915

[36] Feroz AS, Ali NA, Khoja A, Asad A, Saleem S. Using mobile phones to improve young people sexual and reproductive health in low and middle-income countries: a systematic review to identify barriers, facilitators, and range of mHealth solutions. Reprod Health. 2021;18(1):9. doi: 10.1186/s12978-020-01059-7 33453723 PMC7811742

[37] Agarwal S, Glenton C, Tamrat T, Henschke N, Maayan N, Fønhus MS, et al. Decision-support tools via mobile devices to improve quality of care in primary healthcare settings. Cochrane Database Syst Rev. 2021;7(7):CD012944. doi: 10.1002/14651858.CD012944.pub2 34314020 PMC8406991

[38] Hall CS, Fottrell E, Wilkinson S, Byass P. Assessing the impact of mHealth interventions in low-and middle-income countries Á what has been shown to work? 10.3402/gha.v7.25606 PMC4216389 25361730

[39] Kenya National Bureau of Statistics. 2019 Kenya Population and Housing Census. Nairobi: Kenya National Bureau of Statistics. 2019.

[40] Miller LE, Zamudio-Haas S, Otieno B, Amboka S, Odeny D, Agot I. We don’t fear HIV. We just fear walking around pregnant: A qualitative analysis of adolescent sexuality and pregnancy stigma in informal settlements in Kisumu, Kenya. Stud Fam Plann. 2021;52(4):557–70. doi: 10.1111/SIFP.12178 34766351 PMC8932675

[41] Truong HHM, Guzé MA, Kadede K, Amboka S, Otieno B, Odhiambo H, et al. HIV Infection Among Adolescents Residing in Urban Informal Settlements of Kenya. AIDS Educ Prev. 2023;35(3):225–34. doi: 10.1521/AEAP.2023.35.3.225 37410374 PMC10624479

[42] Kenya Bureau of National Statistics. Kenya Demographic and Health Survey 2022. Nairobi, Kenya: Kenya Bureau of National Statistics. 2023.

[43] Ministry of Health D of FH. National Family Planning Guidelines for Health Providers. Nairobi, Kenya: Ministry of Health. 2025. https://www.health.go.ke/sites/default/files/2026-02/Signed%20National%20Family%20Planning%20Guidelines%207th%20Edition%202025%20%281%29.pdf

[44] National AIDS and STI Control Programme (NASCOP), Kenya Medical Research Institute (KEMRI). Guidelines for Conducting Adolescent HIV Sexual and Reproductive Health Research in Kenya. 2015. http://icop.or.ke/wp-content/uploads/2016/10/Adolescents-Guidance-on-HIV-SRH-Research.pdf

[45] Harrington EK, Ouma DC, Pike M, Awuor M, Kimanthi S, Onono M, et al. Exploring Adolescents’ Contraceptive Preferences and Trade-Offs: Findings From a Discrete Choice Experiment in Kenya. Stud Fam Plann. 2025;56(1):41–64. doi: 10.1111/sifp.12280 39780241 PMC12097296

[46] Harrington EK, Hauber B, Ouma DC, Kimanthi S, Dollah A, Onono M, et al. Priorities for contraceptive method and service delivery attributes among adolescent girls and young women in Kenya: a qualitative study. Front Reprod Health. 2024;6:1360390. doi: 10.3389/frph.2024.1360390 38774834 PMC11107089

[47] Harrington EK, Congo O, Kimanthi S, Dollah A, Onono M, Mugo N, et al. PLOS Global Public Health. 2023;3(10):e0001978. doi: 10.1371/journal.pgph.0001978 PMC10602344 37883373

[48] Zia Y, Upadhyay U, Rhew I, Kimanthi S, Congo O, Onono M, et al. Confirmatory Factor Analysis and Validation of the Sexual and Reproductive Empowerment Scale for Adolescents and Young Adults in Kenya. Stud Fam Plann. 2024;55(2):85–103. doi: 10.1111/sifp.12263 38604945 PMC12604660

[49] Fujisaki T, Ouma DC, Awuor M, Aoko C, Kimanthi S, Bukusi EA, et al. Exploring opportunities to enhance the quality of pharmacy-based contraceptive service delivery for adolescents and young women in Kenya: a multimethod qualitative study. BMC Health Serv Res. 2025;25(1):790. doi: 10.1186/s12913-025-12933-0 40462034 PMC12131450

[50] Dam RF, Teo YS. Scamper: How to Use the Best Ideation Methods. Interaction Design Foundation. 2025. https://www.interaction-design.org/literature/article/learn-how-to-use-the-best-ideation-methods-scamper Accessed 2025 December 2.

[51] Dehlendorf C, Fitzpatrick J, Steinauer J, Swiader L, Grumbach K, Hall C, et al. Development and field testing of a decision support tool to facilitate shared decision making in contraceptive counseling. Patient Educ Couns. 2017;100(7):1374–81. doi: 10.1016/j.pec.2017.02.009 28237522 PMC5985808

[52] Witteman HO, Maki KG, Vaisson G, Finderup J, Lewis KB, Dahl Steffensen K, et al. Systematic Development of Patient Decision Aids: An Update from the IPDAS Collaboration. Med Decis Making. 2021;41(7):736–54. doi: 10.1177/0272989X211014163 34148384 PMC8664088

[53] Fisher JD, Fisher WA, Amico KR, Harman JJ. An information-motivation-behavioral skills model of adherence to antiretroviral therapy. Health Psychol. 2006;25(4):462–73. doi: 10.1037/0278-6133.25.4.462 16846321

[54] Ferrer RA, Morrow KM, Fisher WA, Fisher JD. Toward an information-motivation-behavioral skills model of microbicide adherence in clinical trials. AIDS Care - Psychological and Socio-Medical Aspects of AIDS/HIV. 2010. 10.1080/09540121003623719 20552466

[55] Shattuck D, Kerner B, Gilles K, Hartmann M, Ng’ombe T, Guest G. Encouraging contraceptive uptake by motivating men to communicate about family planning: the Malawi Male Motivator project. Am J Public Health. 2011;101(6):1089–95. doi: 10.2105/AJPH.2010.300091 21493931 PMC3093271

[56] Callegari LS, Nelson KM, Arterburn DE, Dehlendorf C, Magnusson SL, Benson SK, et al. Development and Pilot Testing of a Patient-Centered Web-Based Reproductive Decision Support Tool for Primary Care. J Gen Intern Med. 2021;36(10):2989–99. doi: 10.1007/s11606-020-06506-6 33538956 PMC8481447

[57] Dam R, Siang T. Test your prototypes: how to gather feedback and maximize learning. Interaction-design.org. 2023. https://www.interaction-design.org/literature/article/test-your-prototypes-how-to-gather-feedback-and-maximise-learning Accessed 2023 October 29.

[58] Design Kit Methods. https://www.designkit.org/methods.html Accessed 2024 September 8.

[59] Proctor E, Silmere H, Raghavan R, Hovmand P, Aarons G, Bunger A, et al. Outcomes for implementation research: conceptual distinctions, measurement challenges, and research agenda. Adm Policy Ment Health. 2011;38(2):65–76. doi: 10.1007/s10488-010-0319-7 20957426 PMC3068522

[60] Communications Authority of Kenya. Mobile, internet, and tech services surge in Kenya as digital shift accelerates. 2025. https://www.ca.go.ke/mobile-internet-and-tech-services-surge-kenya-digital-shift-accelerates. 2025. Accessed 2025 December 3.

[61] Ferretti A, Adjei KK, Ali J, Atuire C, Ayuk BT, Banougnin BH, et al. Digital tools for youth health promotion: principles, policies and practices in sub-Saharan Africa. Health Promotion International. 2024;39(2):daae030. doi: 10.1093/heapro/daae030 38558241 PMC10983781

[62] Cuadros DF, Kiragga A, Tu L, Awad S, Bwanika JM, Musuka G. Unpacking social and digital determinants of health in Africa: a narrative review on challenges and opportunities. Mhealth. 2025;11:41. doi: 10.21037/MHEALTH-24-73/COIF PMC12314699 40755923

[63] Stevens R, Machiyama K, Mavodza CV, Doyle AM. Misconceptions, misinformation, and misperceptions: a case for removing the “mis-” when discussing contraceptive beliefs. Stud Fam Plann. 2023;54(1):309–21. doi: 10.1111/SIFP.12232 36753058

[64] Chipako I, Singhal S, Hollingsworth B. Impact of sexual and reproductive health interventions among young people in sub-Saharan Africa: a scoping review. Front Glob Womens Health. 2024;5:1344135. doi: 10.3389/fgwh.2024.1344135 38699461 PMC11063325

[65] Mwaisaka J, Gonsalves L, Thiongo M, Waithaka M, Sidha H, Agwanda A. Exploring contraception myths and misconceptions among young men and women in Kwale County, Kenya. BMC Public Health. 2020;20(1). doi: 10.1186/S12889-020-09849-1 33176738 PMC7661170

[66] Jonas K, Duby Z, Maruping K, Harries J, Mathews C. Rumours, myths, and misperceptions as barriers to contraceptive use among adolescent girls and young women in South Africa. Front Reprod Health. 2022;4:960089. doi: 10.3389/frph.2022.960089 36406890 PMC9673823

[67] Gichangi P, Gonsalves L, Mwaisaka J, Thiongo M, Habib N, Waithaka M, et al. Busting contraception myths and misconceptions among youth in Kwale County, Kenya: results of a digital health randomised control trial. BMJ Open. 2022;12(1):e047426. doi: 10.1136/bmjopen-2020-047426 34992099 PMC8739061

[68] Gonsalves L, Kamuyango A, Chandra-Mouli V. Pharmacies: an important source of contraception for some adolescents, but not a panacea for all. Sex Reprod Health Matters. 2023;31(1):2221883. doi: 10.1080/26410397.2023.2221883 37417799 PMC10332186

