# Supplementary material for: Co-design of a digital health intervention to provide pharmacy-based, person-centered contraceptive decision support for youth in Kenya
Source: PLOS Glob Public Health. 2026 Sep 17;6(9):e0007335. doi: 10.1371/journal.pgph.0007335 (PMC13585247; doi:10.1371/journal.pgph.0007335)
Supplement: Supplementary file 1 [file pgph.0007335.s001.pdf]

Co-design of a digital health intervention to provide pharmacy-based, person-centered contraceptive decision support for youth in Kenya

Structured Reflexivity Statement guided by: *Morton B, Vercueil A, Masekela R, Heinz E, Reimer L, Saleh S, et al. Consensus statement on measures to promote equitable authorship in the publication of research from international partnerships. Anaesthesia. 2022;77(3).*

**Study conceptualisation**	
1. How does this study address local research and policy priorities?	Adolescent sexual and reproductive health, including contraception to prevent undesired pregnancy, is recognized as a key priority in the Kenya Ministry of Health
2. How were local researchers involved in study design?	Dr. Gitome and Prof Bukusi conceptualized the study with Dr. Harrington. Dr. Gitome and Mr. Cheruiyot led the human-centered design process including data collection and interpretation.
**Research management**	
3. How has funding been used to support the local research team?	The majority of funding (>90%) from the funding sources went to support the local research team. Funding for Kenyan researchers has been provided for salary support, transportation, study-related activities, and conference attendance for an early career investigator from the team to present his work at a panel at the 2025 FIGO Conference in Cape Town, South Africa.
**Data acquisition and analysis**	
4. How are research staff who conducted data collection acknowledged?	Dr. Gitome is senior author and directed all data collection in collaboration with Nelson Cheruiyot (co-author). Merceline Awuor (study coordinator) and Cellestine Aoko are co-authors; they co-facilitated data collection and data management.
5. How have members of the research partnership been provided with access to study data?	Kenya-based co-investigators named on the IRB (Bukusi, Ouma, Gitome, Cheruiyot) had access to the study data through a secure study drive, as did Merceline Awuor and Cellestine Aoko.
6. How were data used to develop analytical skills within the partnership?	All members of the research partnership gained familiarity with human-centered design methods through weekly team calls, workshop facilitation, analysis retreats, and the writing process.
**Data interpretation**	

Co-design of a digital health intervention to provide pharmacy-based, person-centered contraceptive decision support for youth in Kenya

7. How have research partners collaborated in interpreting study data?	Study data were discussed and interpreted during weekly team calls, which included members of the Kenya-based and US-based research team.
**Drafting and revising for intellectual content**	
8. How were research partners supported to develop writing skills?	Dr. Ouma completed a public health degree at the University of Washington during the project time period, which includes an intensive course supporting research writing.
9. How will research products be shared to address local needs?	Results will be presented to the Kisumu County Health Management Team along with the feasibility study in 2026. The published manuscript will be disseminated to the adolescent sexual and reproductive health team lead at the Kenya Ministry of Health.
**Authorship**	
10. How is the leadership, contribution, and ownership of this work by LMIC researchers recognized within the authorship?	Dr. Gitome is recognized as senior author. Among ten co-authors in this multidisciplinary paper featuring three UW departments, Kenya-based human-centered design researchers, and local study staff, six are Kenyan nationals who live and work in Kenya.
11. How have earlier career researchers across the partnership been included within the authorship team?	Dr. Ouma is an early career researcher who was based at KEMRI at the time this work was completed. Dr. Gitome is a mid-career researcher based at KEMRI.
12. How has gender balance been addressed within the authorship?	Seven authors identify as cisgender women, and three authors identify as cisgender men. Our partnership did not specifically address gender balance in the authorship team.
**Training**	
13. How has the project contributed to training of LMIC researchers?	Dr. Gitome and Nelson Cheruiyot trained all team members, including Kenyan team members, in human centered design methods. Dr. Ouma was supported in presenting at an international conference.
**Infrastructure**	
14. How has the project contributed to improvements in local infrastructure?	This project has not contributed to infrastructure improvements other than

	contributing to the maintenance of research facilities through monthly fees.
**Governance**	
15. What safeguarding procedures were used to protect local study participants and researchers?	From an ethical perspective, we used a variety of procedures and best practices to protect adolescent women's privacy and confidentiality, such as keeping study inclusion criteria private, and community mobilization through peers. Our team used the appropriate channels for regulatory input and ethical approval to protect participants. KEMRI Scientific Ethics Research Unit (KEMRI/SERU/CMR/4917) and the University of Washington Human Subjects Division (00020507) granted ethical approval for this study, as well as a waiver for parental permission for minors. This study was also approved by the Kenya National Commission for Science, Technology, and Innovation (NACOSTI, research permit P/24/33897) and the Kisumu County Health Management Team authorized the conduct of the study in Kisumu County. Written informed consent (age 18 years or older) or assent (age 15-17 years) was obtained from all participants.
